# Practical Implementation of Emergent After-Hours Radiation Treatment Process Using Remote Treatment Planning on Optimized Diagnostic CT Scans

**DOI:** 10.7759/cureus.33100

**Published:** 2022-12-29

**Authors:** Kareem R Fakhoury, Leah K Schubert, Mychaela D Coyne, Wes Aldridge, Sabrina Zeiler, Kelly Stuhr, Timothy V Waxweiler, Tyler P Robin, Tracey E Schefter, Brian D Kavanagh, Sameer K Nath

**Affiliations:** 1 Department of Radiation Oncology, University of Colorado School of Medicine, Aurora, USA

**Keywords:** process improvement, palliative radiation, after hours, diagnostic ct, radiation oncology, emergency planning

## Abstract

The purpose of this report is to present the implementation of a process for after-hours radiation treatment (RT) utilizing remote treatment planning based on optimized diagnostic computed tomography (CT) scans for the urgent palliative treatment of inpatients. A standardized operating procedure was developed by an interprofessional panel to improve the quality of after-hours RT and minimize the risk of treatment errors. A new diagnostic CT protocol was created that could be performed after-hours on hospital scanners and would ensure a reproducible patient position and adequate field of view. An on-call structure for dosimetry staff was created utilizing remote treatment planning. The optimized CT protocol was developed in collaboration with the radiology department, and a novel order set was created in the electronic health system. The clinical workflow begins with the radiation oncologist notifying the on-call team (therapist, dosimetrist, and physicist) and obtaining an optimized diagnostic CT scan on a hospital-based scanner. The dosimetrist remotely creates a plan; the physicist checks the plan; and the patient is treated. Plans are intentionally simple (parallel opposed fields, symmetric jaws) to expedite care and reduce the risk of error. Education on the new process was provided for all relevant staff. Our process was successfully implemented with the use of an optimized CT protocol and remote treatment planning. This approach has the potential to improve the quality and safety of emergent after-hours RT by better approximating the normal process of care.

## Introduction

Emergent after-hours radiation therapy (RT) for oncologic emergencies presents unique clinical challenges for the safe delivery of care. Positioning, planning, and treatment often occur manually and quickly. These patients may also require a higher level of care due to life- or organ-threatening symptoms [1]. Since radiation therapy (RT) is primarily delivered in the outpatient setting, standard clinical resources are often unavailable after-hours. In particular, computed tomography (CT) simulation may not be obtainable because it is not practically feasible for all on-call therapists to be trained to operate the scanners. As such, the on-call care team will typically perform a linear accelerator-based simulation and treatment utilizing manual dose calculations with simple beam arrangements.

Given the condensed timeline and complexity of care, there is a higher risk of treatment errors after hours [2,3]. Without the anatomical information of CT simulation or the accuracy of computer-based treatment planning systems (TPS), the clinical treatment approach is more prone to targeting and planning errors [2]. Furthermore, clinical experience with bony anatomy landmarks based on portal imaging has decreased with the advent of CT simulation. This knowledge gap, coupled with the infrequency of after-hours treatments, leads to a higher risk for mistreatment.

Several institutions have shown the feasibility and accuracy of planning RT on diagnostic CT scans [4-6]. Because diagnostic CT scanning is readily available for hospitalized patients on weekends and after-hours, diagnostic scan-based planning (DSBP) may provide an opportunity to improve treatment planning for emergent cases. However, DSBP requires a scan with an adequate field of view (FOV) and reproducible patient position [5,6], as well as a dosimetrist or physicist to create a treatment plan. While remote RT planning has been reported in the past [7-9], it became common during the SARS-CoV-2 pandemic [10,11]. As experience with remote planning increased, it became feasible to staff an on-call dosimetrist.

In an effort to improve the quality and safety of emergent RT delivered after-hours, we designed an institutional standard operating procedure (SOP) for DSBP with remote treatment planning. Herein we describe the practical clinical implementation of this approach, including the development of an optimized diagnostic CT protocol and staffing an on-call remote dosimetrist.

## Technical report

Process development

To create a standardized approach, we assembled an interprofessional panel involving lead radiation oncologists, physicists, dosimetrists, therapists, and nurses to design a process. Using reports from our incident learning system and expert opinion, several areas of concern for potential errors during after-hours treatment were identified, including target localization, misalignment, and manual dose calculations. The panel then created a consensus best-practice SOP utilizing DSBP with remote treatment planning, which would allow target localization on cross-sectional imaging, fusion to onboard imaging for accurate alignment, and more accurate estimation of three-dimensional dose distributions.

Optimization of diagnostic CT scans

**Figure 1:**
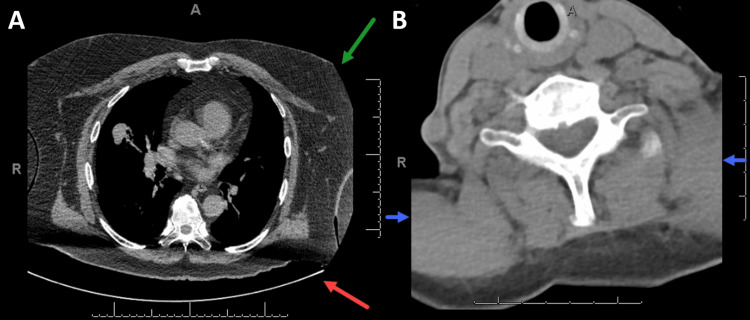
(A) Diagnostic CT scan of the chest showing a curved tabletop (red arrow) limiting reproducibility of patient position and narrow FOV cropping the body (green arrow) limiting the ability to use fields that pass through this area. (B) Diagnostic CT scan of the cervical spine showing a narrow FOV which crops the body (blue arrows) and prevents the ability to use parallel opposed fields. FOV: field of view

The panel reviewed the available literature and utilized expert opinion to identify potential challenges using diagnostic CTs for radiation planning. In order to use a diagnostic CT scan for RT planning, the patient must be positioned in a reproducible way. During diagnostic scans, patients are often oriented feet-first with arms above their head in a non-indexed fashion, in contrast to the standard RT position, which is usually head-first with arms at the side. A curved tabletop is standard for diagnostic CTs, which may lead to differences in positioning and anatomy when subsequently using a flat tabletop during RT (Figure 1A). Furthermore, there must be full inclusion of the treatment target volume on the diagnostic imaging, which may be cropped when scanning by standard diagnostic body subsites (e.g., chest, pelvis, spine). Finally, the narrower FOV used in diagnostic CT scans may crop parts of the body, preventing certain beam arrangements and/or accurate dose calculations if soft tissue is missing at the entrance of the beam (Figure 1B).

Rather than relying on an existing diagnostic CT scan with its inherent limitations, we developed a new diagnostic CT scanning protocol that would be ordered at the time of consultation and would be optimized for treatment planning (Table 1). To maximize the likelihood of reproducible patient positioning, the protocol specifies that patients are positioned on a flat tabletop insert with arms at the side. The only devices used are a knee sponge for patient comfort and a head sponge that approximates one of the head support devices used in the radiation oncology department. The protocol specifies a wide FOV of 60 cm and a scanning area from the vertex through mid-femur. The slice thickness was set to 3 cm with a pitch of 1. In the free text of the order, the radiation oncologist has the option to change any of the above parameters as clinically indicated. There are options for including intravenous (IV) contrast or a formal scan interpretation by the radiology department.

**Table 1:** Challenges posed by the use of typical diagnostic CT scans for RT planning and how they were modified in the optimized diagnostic CT protocol. FOV: field of view; TPS: treatment planning system

Typical Diagnostic CT	Challenge Posed	Optimized Diagnostic CT
Feet-first	Must re-orient image in TPS	Head-first
Arms above head	Non-indexed, variability in positioning	Arms at side
Head holder attached to end of table	Posterior displacement of neck	Standardized head sponge
Curved tabletop	Difference in outer body contour on treatment table	Flat tabletop insert
Narrow FOV	Cropped body	Wide FOV
Scan per body site	Cropped treatment target	Scan vertex to mid-femur

Remote treatment planning

As a result of the SARS-CoV-2 pandemic, our dosimetry staff acquired the equipment and experience required for remote treatment planning using Eclipse v.15.6 (Varian Medical Systems, Palo Alto, CA). The system could be accessed through a cloud server (Citrix Systems, Ft. Lauderdale, FL) or by remotely accessing an individual workstation using a virtual private network. Prior to the establishment of this process, on-call staff included one therapist, one physicist, and attending and resident radiation oncologists. Because of the flexibility afforded by remote access to the TPS, it became feasible to create an on-call structure for our dosimetry staff to allow DSBP over the weekend.

The emergent after-hours treatment process

**Figure 2:**
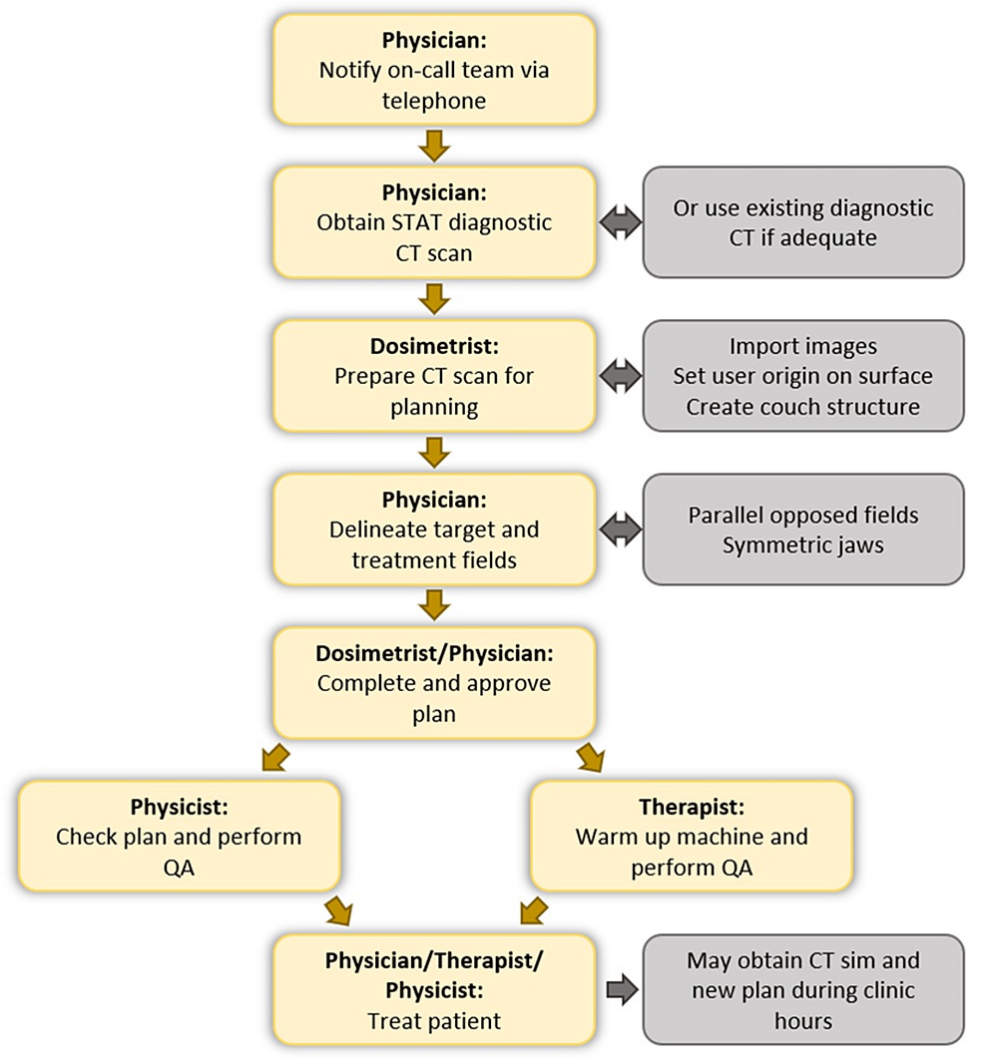
Workflow for emergent after-hours treatments.

**Figure 3:**
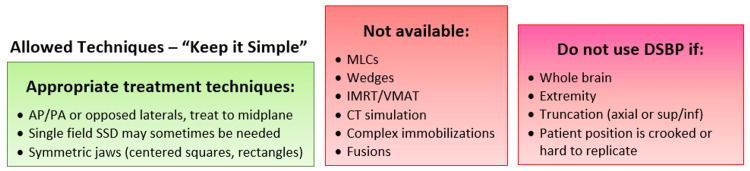
A visual guide included in our treatment process to indicate appropriate treatment techniques, as well as circumstances under which DSBP should not be used. AP/PA: anteroposterior/posteroanterior; DSBP: diagnostic scan-based planning; IMRT: intensity-modulated radiation therapy; MLC: multileaf collimator; SSD: source-to-skin distance; sup/inf: superior/inferior; VMAT: volumetric modulated arc therapy

The process for emergent after-hours treatments was designed as outlined in Figure 2 and made directly available on the call schedule. When the clinical decision to provide urgent treatment is made, the physician notifies the rest of the on-call team (therapist, physicist, dosimetrist) using telephone communication. The physician determines whether there is an existing diagnostic CT scan that is appropriate for DSBP; if not, an optimized diagnostic CT is ordered with urgent priority (i.e., STAT). Once the scan is available, the dosimetrist remotely imports the images, sets the user origin based on a surface anatomical landmark (e.g., suprasternal notch or umbilicus), and adds a structure to approximate the treatment table. The physician then delineates the target and treatment fields, and the dosimetrist completes the plan. The process specifies that only simple techniques with parallel opposed fields and symmetric jaws are to be used (Figure 3). The physicist checks the plan before the patient is treated. Depending on the number of fractions and desired dose conformality, the physician decides to continue with the DSBP (preferred) or obtain a formal CT simulation to create a new treatment plan during regular clinic hours.

Clinical implementation

**Figure 4:**
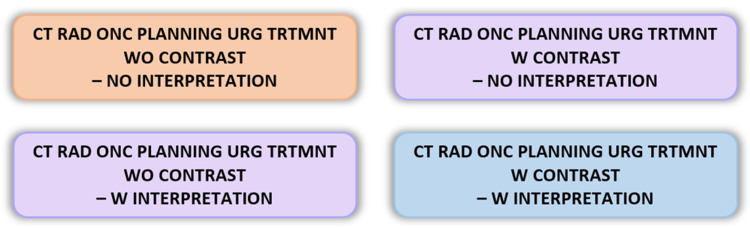
Optimized CT order names based on presence or absence of IV contrast and formal radiology interpretation. RAD ONC: radiation oncology; URG TRTMNT: urgent treatment; W: with; WO: without

The optimized CT protocol was formalized by the radiology department, and orders were created in our electronic health system (Epic Systems, Verona, WI) (Figure 4). Education on the new process was provided for all relevant staff, including therapists, physicists, dosimetrists, and physicians as well as for radiology staff. It was emphasized to staff that plans are intentionally made to be simple, without multileaf collimation, wedges, or image fusion (unless absolutely necessary), to reduce complexity and expedite treatment planning.

## Discussion

After-hours oncologic emergencies amenable to RT occur in challenging resource-limited settings [1,12]. In this study, we present our experience developing an SOP utilizing optimized diagnostic CTs for radiation planning for the treatment of oncologic emergencies after-hours. Our expert panel identified potential safety concerns in advance of implementation and created a consensus workflow with interprofessional feedback from therapists, dosimetrists, physicists, and radiologists. Overall, we successfully developed and implemented our DSBP program with the intention of reducing the risk of mistreatment during the high-pressure setting of urgent after-hours treatment. We also provide details of our protocols for other institutions that may wish to implement a similar process.

There are only limited published manuscripts on this topic, primarily focusing on dosimetry. In particular, prior studies have shown that there are differences in dose calculations between CT simulation planning and DSBP due to variability in patient positioning and CT numbers between machines. However, these differences are small (± 1%-4%) and unlikely to be clinically meaningful [4-6]. Our study is unique in that we focus on the practical implementation of a reliable workflow utilizing DSBP.

There are several limitations to this approach. Within our hospital network, fewer than 20 patients received after-hours emergent treatments from July 2020 to July 2021. The relatively low frequency of these events may limit the practicality of wide adoption, though some institutions have reported treating 6 or more patients per month [1,12]. The process document was made directly available as a link from the on-call schedule for ease of access and compliance. Furthermore, while we can use electronic portal imaging and cone beam CT to align the patient, we have not incorporated surface-guided RT due to concerns that it would add additional complexity and alignment issues that may delay treatment.

Given the practical benefits of DSBP, future work may show benefits to extending this approach to non-emergent treatments [13]. Particularly for palliative RT in patients with limited functional status or logistical barriers (e.g., transportation, distance from the treatment center, hospice enrollees), the ability to treat without a CT simulation provides an opportunity to expedite treatment, limit visits, and reduce costs. As such, our next project on this topic will focus on quantifying potential improvements in patient-centric outcomes.

## Conclusions

A novel process for emergent after-hours RT delivery was successfully implemented. This process relied on the use of an optimized CT protocol and remote treatment planning. This approach has the potential to improve the quality and safety of emergent after-hours RT by better approximating the normal process of care. Future work in this area may show benefits to extending DSBP to non-emergent treatments, including palliative RT for patients with limited functional status and logistical barriers, who would benefit from expedited treatment, limited visits, and reduced costs.
